# The Use of Flow Diversion for the Treatment of Intracranial Aneurysms: Expansion of Indications

**DOI:** 10.7759/cureus.472

**Published:** 2016-01-28

**Authors:** Adam M Brouillard, Xingwen Sun, Adnan H Siddiqui, Ning Lin

**Affiliations:** 1 Department of Neurosurgery, Jacobs School of Medicine and Biomedical Sciences, University at Buffalo, State University of New York, Buffalo, New York; 2 Department of Neurosurgery, Weill Cornell Medical College; 3 New York Presbyterian Hospital

**Keywords:** intracranial aneurysm, endovascular treatment, flow diversion, ruptured aneurysm, coil embolization, complication

## Abstract

Flow diversion is a novel concept for treating anatomically challenging intracranial aneurysms and has gained increasing acceptance. Flow diverter stents, such as the Pipeline Embolization Device (PED) (ev3-Covidien, Irvine, CA, USA), are approved for treating unruptured large and giant aneurysms from the internal carotid artery between the superior hypophyseal and cavernous segments. However, technological advances and recent clinical results suggest that flow diversion can be safely and effectively used in treating ruptured aneurysms, posterior circulation aneurysms, and distal anterior circulation aneurysms. In this brief review, we aim to investigate the recent evidence on the utilization of PEDs in these controversial vascular territories and to discuss whether the indications for flow diversion can be expanded.

## Introduction and background

The use of the Pipeline Embolization Device (PED) (ev3-Covidien, Irvine, CA, USA) as a flow diverter stent for the treatment of intracranial aneurysms has gained increasing acceptance during the past several years. Despite its relatively short time in clinical utilization, the PED has demonstrated unique versatility in treating anatomically complex aneurysms that are technically challenging for conventional microsurgical or endovascular therapies [1-13]. The PED is a self-expanding, braided cylindrical mesh consisting of 48 strands of cobalt-chromium and platinum-tungsten wires, each of which is 28-33 mm in diameter. Unique to an endoluminal device, such as the PED, is the 30-35% metal surface area coverage that far exceeds the 6.5-9.5% coverage for regular intracranial stents designed to assist endosaccular embolization [1, 14]. This low-porosity feature allows the PED to alter the hemodynamics within the parent vessel and the aneurysm sac by reducing the inflow rate and average wall shear stress and subsequently to induce aneurysm thrombosis and occlusion [3, 15]. It is postulated that gradual endothelialization of the device results in reconstruction of the aneurysm neck and resorption of the thrombus, thus, eliminating the mass effect of an aneurysm [3]. Furthermore, the ability to telescope multiple PED stents to lengthen the endoluminal construct enhances the versatility of the device and the radiopacity of the platinum component allows for effective visualization under fluoroscopic imaging [3, 14]. After the publication of promising results from large multicenter clinical trials [7, 16], the U.S. Food and Drug Administration approved the utilization of the PED for the treatment of large or giant wide-necked aneurysms from the petrous to the superior hypophyseal segments of the internal carotid artery (ICA).

Compared with conventional endosaccular embolization with coils alone or coils in combination with stents, flow diverters can provide better neck reconstruction and reduce the rate of long-term recanalization and the need for retreatment [17]. Their use is also associated with lower procedural cost [18]. These advantages can theoretically be applied to ruptured aneurysms and to aneurysms arising from vessel segments other than the cavernous and paraclinoidal ICA, such as those of the posterior circulation or the distal anterior circulation beyond the ICA terminus. In this brief review, we aim to investigate recent evidence on the utilization of PEDs in these controversial vascular territories and to discuss whether the indications for flow diversion can be expanded. 

### Evolution of flow diversion           

While PED is the most widely utilized flow diverter stent in the U.S., several other flow diversion devices have been clinically available or under investigation. The SILK device (Balt Extrusion; Montmorency, France) was the first flow diversion stent available for clinical use and received the CE Mark in 2008. It is a flexible, self-expanding braided mesh cylinder composed of 48 nickel-titanium (nitinol) strands and provides 35-55% of metal coverage [14]. Berge, et al. reported their findings with the Silk stent in 77 aneurysms and found a 7.8% morbidity and 3% mortality at one-year follow-up [19]. Delayed complications, such as thromboembolic events or delayed aneurysm rupture, occurred in 10.9% of patients [19]. Additionally, Lubicz, et al. analyzed 34 aneurysms treated with the Silk device and found a 4% mortality rate and 15% morbidity rate. Parent artery stenosis was observed in 33% of the patients at six-month follow-up [5]. 

The Surpass Flow Diverter (Stryker Neurovascular, Fremont, CA, USA) is considered a new generation of endoluminal device and is currently undergoing review in the Safety and Effectiveness of an Intracranial Aneurysm Embolization System for Treating Large or Giant Wide Neck Aneurysms (SCENT) Trial (https://clinicaltrials.gov/ct2/show/NCT01716117). The stent is a self-expanding tubular mesh made of Cobalt-chromium with 30% metal coverage [14, 20]. The stent design emphasizes achieving constant pore density over various diameters of the device, and as a result, the 2.5 mm diameter device has 48 Cobalt-chromium strands, whereas the 3 and 4 mm devices have 72 wires and the 5 mm device has 96 [14, 20]. In a series published by De Vries, et al., the investigators used Surpass to treat 37 patients, with occlusion rates of 94% at non-bifurcation sites and 50% at bifurcation locations. One patient suffered a stroke following treatment; no mortality was reported [20]. 

Additionally, the Flow Redirection Endoluminal Device (FRED) (MicroVention, Tustin, CA, USA) has also been investigated (Pivotal Study of the FRED Stent System in the Treatment of Intracranial Aneurysms; https://clinicaltrials.gov/ct2/show/NCT01801007). The FRED has a novel design that includes a dual-layer composition with a low-porosity inner mesh and high-porosity outer stent [21]. The outer stent decreases friction on the parent vessel during microcatheter navigation and stent delivery, whereas the high metal-coverage inner mesh diverts flow away from an aneurysm [21]. Moreover, the FRED can be resheathed completely with up to 50% of the stent deployed. Kocer, et al. utilized the FRED for 37 cases (35 unruptured and two previously ruptured) in which one device was utilized per case [21]. The authors report a 3% complication rate with no mortality or morbidity. No follow-up is available beyond one year; thus, long-term results, as well as evaluation of the FRED in ruptured cases, remain unclear. Further development and analysis of these newly developed flow diverters may yield more effective treatment strategies and more favorable outcomes for patients.

## Review

### Expansion of indications

The PED relies on the concept of flow diversion to treat aneurysmal lesions without the benefit of packing aneurysm domes with coils or other embolic materials. Flow diversion refers to the endoluminal reconstruction of the vessel instead of endosaccular filling to induce thrombosis [14]. The parent artery is reconstructed mechanically by the disruption of blood flow and reduction of shear stress within the aneurysm dome [3, 8]. Flow diversion leads to natural thrombosis by facilitated stasis within an aneurysm that is later reabsorbed while the PED construct is endothelialized to seal the artery [3]. As a result, close follow-up care is essential after flow diversion treatment as it may take up to 12 months for the parent vessel to fully heal and likely longer for the thrombus to be reabsorbed [14]. Comparatively, traditional endosaccular treatment does not rely on hemodynamic alteration and is limited by the density of coil packing, difficulty in giant and complicated aneurysm morphology, and the lack of parent vessel repair [7]. The investigators of the Pipeline for the Intracranial Treatment of Aneurysms (PITA) trial concluded that there was a greater occlusion rate with the PED than conventional endosaccular treatment, irrespective of aneurysm size or morphology [7]. Lanzino, et al. conducted a direct comparison between the PED and standard endovascular treatment with 22 matched paraclinoid ICA aneurysms and found complete occlusion in 76% of PED cases compared to only 21% in coiling cases [22]. Furthermore, the utility of the PED is enhanced by its ability to be implanted in regions containing vessel branches. Perforators can typically remain patent after PED treatment along the parent vessel if metal coverage does not exceed 50% at the perforator orifice, which is typically the threshold above which blood flow to a perforator branch begins to diminish to a meaningful extent [3].

Recent literature suggests that utilization of the PED can be expanded well beyond its initial indication for large and giant aneurysms from the ICA between the superior hypophyseal and cavernous segments. For example, case-controlled studies compare PED treatment favorably to coil embolization for small, as well as large, anterior circulation aneurysms, thus, supporting PED usage for aneurysms less than 10 mm in size [17, 23]. Additionally, the PED device can be applied to ruptured aneurysms, posterior circulation aneurysms, distal anterior circulation aneurysms, and blister aneurysms, as discussed below.  

Ruptured Aneurysms

Use of the PED in ruptured cases was initially avoided owing to the belief that it would be ineffective in preventing short-term morbidity and mortality in the setting of recent hemorrhage. However, several reports have indicated that these limits are not absolute and ruptured and dissecting aneurysms may benefit from PED treatment [9-13]. Lin, et al. reported a series of 26 ruptured cases treated at five participating treatment centers using the PED [24]. PED deployment was successful in all cases, and adjunctive coiling was used in 12 cases to induce immediate thrombosis. Periprocedural complications occurred in 19.2% of cases with three in-hospital deaths. Aneurysms in 18 of 23 (78.2%) patients for whom follow-up was available were completely occluded as documented by angiography. In line with this study, Chan, et al. published a series consisting of eight patients with ruptured dissecting aneurysms [25]. Technical success was achieved in all patients. Follow-up angiography at six months showed complete aneurysm occlusion in all patients. Two patients experienced symptomatic complications. There were no procedure-related thrombotic or rebleeding events [25]. Another study conducted by Chalouhi, et al. suggested again the efficacy and safety of the PED utilization in ruptured aneurysms [26]. In this series of 20 ruptured aneurysms treated at two centers, complete occlusion was achieved in 80% of the cases and partial occlusion in the other 20%. A procedure-related complication occurred in only one case in which the aneurysm dome ruptured during adjunctive coil deployment. Favorable outcome (modified Rankin scale score 0-2) was reported in 95% of the patients at the last follow-up date. The PED is particularly efficacious in the treatment of blister aneurysms as these lesions are too small (2-3 mm) to be effectively clipped or coiled. PED treatment allows the vessel to heal while diverting flow away from these dangerous lesions. In the report by Lin, et al., all eight blister cases were completely occluded with favorable outcomes achieved in seven of these cases [24]. These reports demonstrate the safety and effectiveness of PED treatment and the potential for its application in a variety of ruptured aneurysm situations.

Posterior Circulation Aneurysms: 

While endovascular coiling has long been considered safe and effective in treating posterior circulation aneurysms, early experience of PED in posterior circulation aneurysms has been reported to incur significant morbidity and mortality, with some series describing mortality rates close to 50% [27]. The potential sources of complications included overlapping stents causing brainstem perforator thrombosis, lack of coil usage resulting in delayed aneurysm rupture, and unsuccessful antiplatelet therapy. However, with the accumulation of clinical experience, technical success has been increasingly reported. Munich, et al. described a series of 11 posterior circulation fusiform aneurysms successfully treated with PED, and 90% of the aneurysms were completely occluded as demonstrated by follow-up angiography [28]. With additional attention to antiplatelet therapy and perforating artery anatomy, the authors were able to achieve a significantly lower complication rate by comparison with previous reports. Three patients experienced postoperative complications, and one patient died of pulmonary complications following hemiparesis. Two suffered from new neurological deficits that were significantly improved at the time of follow-up. Albuquerque, et al. reported a series of 17 cases of posterior circulation aneurysms treated with PED [29]. Follow-up angiographic studies showed complete occlusion in 14 of 17 patients and near-complete occlusion (> 90%) in all cases. Only one (5.9%) case of a complication related to the development of a parenchymal hematoma after ventriculostomy replacement was reported in this series. The authors [29] concluded that the complication rate could be reduced by close monitoring of platelet inhibition status and careful patient selection (with exclusion of dolichoectatic and giant fusiform aneurysms, which were previously shown to be associated with a high risk of complications [27]). Taken together, these data suggest that PED could be a viable option in addition to the traditional surgical and endovascular management of posterior circulation aneurysms.

Distal Anterior Circulation Aneurysms:

In the past, the PED was not widely used in the treatment of distal anterior circulation aneurysms due to their relatively superficial anatomy and good accessibility for microsurgical clipping. Placement of PEDs at the A1 segment of the anterior cerebral artery and the M1 segment of the middle cerebral artery was believed to increase the risk of perforator stroke because of the richness of lenticulostriate branches. The delivery of the device in the relatively small caliber vessels of the distal anterior circulation was also considered to be technically difficult. However, clinical experience with such potential complications has rarely been published. Puffer, et al. reported four ophthalmic artery occlusions in a series of 19 patients with paraclinoid ICA aneurysms [30]. However, none of the patients developed visual symptoms in the long-term follow-up. In a recent multicenter study conducted by Lin, et al., 27 of 28 patients with distal anterior aneurysms, including 15 fusiform aneurysms, five dissecting aneurysms, and eight saccular aneurysms, were successfully treated with PED. Adjunctive coiling was used in six cases. The average size of these aneurysms was 12.3 mm and seven were giant. Perioperative complications occurred in three cases (10.7%), including a single case of stroke due to device failure. Complete occlusion was achieved in 21 of 27 aneurysms (77.8%), and 27 of 28 (96.4%) patients had a favorable clinical outcome (modified Rankin scale score 0-2) [31]. These results collectively indicate that PED can be safely delivered and deployed in relatively small vessels in the distal anterior circulation with a high success rate. Using a single, long PED could ease the placement process and avoid perforator occlusion.

### Complications associated with the Pipeline Embolization Device

Several reports have described both ischemic and hemorrhagic complications that can occur during or after PED treatment. Van Rooij and Sluzewski reported a case of an anterior cerebral artery aneurysm that was treated with two telescoped PEDs [32]. A post-procedure infarction in the left basal ganglia from the A1 segment resulted in cognitive impairment and memory dysfunction in the patient. Imaging revealed that the PED construct had occluded perforator arteries due to an elevated surface area from telescoping. Another case of late thrombosis (> one-year post-treatment) of a PED placed in the vertebral artery was also reported [33]. Adjunctive coiling was used in the initial treatment and the etiology of the thrombosis was unexplained, although the authors postulated that inadequate platelet inhibition was likely the cause. In a series of 28 distal anterior circulation aneurysms, Lin, et al. reported two periprocedural ischemic strokes (one related to a device failure and one delayed stroke due to perforator thrombosis) [31]. These data suggest that while PED-related perforator occlusion has rarely been reported, it is a complication that must be accounted for during treatment of aneurysms located in the perforator-rich areas of A1, M1, and posterior circulation vessels. This risk could be reduced by placing a single, long flow diverter stent and avoiding telescoping of multiple devices along the perforator-rich segments.

Delayed aneurysm rupture after PED placement has been reported by multiple investigators [34-35], which may result from the lack of immediate dome protection by flow diversion treatment and mural destabilization within the aneurysm dome [34]. This type of complication could be minimized by using coils in conjunction with PEDs for aneurysms that are considered fragile with imminent danger of hemorrhage, such as posterior circulation aneurysms, dissecting aneurysms, and ruptured aneurysms [36]. Ipsilateral distal intraparenchymal hematoma after PED placement has also been reported, which was thought to have been caused by the hemorrhagic conversion of embolic strokes during PED deployment, especially in the setting of dual platelet therapy [37]. Another significant complication of PED usage in a ruptured case was noted by McTaggart, et al., in which a ruptured dissecting aneurysm of the right vertebral artery and the right posterior inferior cerebellar artery was treated [38]. Vasospasm noted five days post-procedure resulted in PED retraction in the constricted vessel. A second PED was placed due to the migration of the first stent; however, vasospasm significantly complicated the positioning of this device. The patient successfully returned to neurological baseline level after the second procedure. The authors noted that in addition to the pitfalls of antiplatelet therapy, delayed retraction and difficulty in secondary stent positioning can be expected if vasospasm occurs in a ruptured case [38]. Administration of nimodipine may be beneficial in these cases to reduce the possibility of vasospasm-related complications.

## Conclusions

The use of flow diversion for the treatment of intracranial aneurysms has yielded favorable clinical results and represents a paradigm shift for the management of large, fusiform aneurysms. Besides the original indication, the PED can be utilized safely and effectively for ruptured aneurysms, blister aneurysms, and aneurysms located in the posterior circulation or distal anterior circulation that are difficult to treat with conventional clipping or coiling. The available results of the Surpass and the FRED are encouraging, yet relatively limited. Larger-scale studies with long-term follow-up data are needed to further examine the safety profile and durability of the new devices as well as to afford opportunities for outcome improvement.
